# Flavivirus recruits the valosin-containing protein–NPL4 complex to induce stress granule disassembly for efficient viral genome replication

**DOI:** 10.1016/j.jbc.2022.101597

**Published:** 2022-01-19

**Authors:** Masashi Arakawa, Keisuke Tabata, Kotaro Ishida, Makiko Kobayashi, Arisa Arai, Tomohiro Ishikawa, Ryosuke Suzuki, Hiroaki Takeuchi, Lokesh P. Tripathi, Kenji Mizuguchi, Eiji Morita

**Affiliations:** 1 Department of Biochemistry and Molecular Biology, Faculty of Agriculture and Life Science, Hirosaki University, Hirosaki, Japan; 2 Division of Biomolecular Function, Bioresources Science, United Graduate School of Agricultural Sciences, Iwate University, Morioka, Japan; 3 Laboratory of Intracellular Membrane Dynamics, Graduate School of Frontier Biosciences, Osaka University, Osaka, Japan; 4 Department of Genetics, Graduate School of Medicine, Osaka University, Osaka, Japan; 5 Laboratory of Viral Infection, Research Institute for Microbial Diseases, Osaka University, Osaka, Japan; 6 Department of Microbiology, Dokkyo Medical University School of Medicine, Tochigi, Japan; 7 Department of Virology II, National Institute of Infectious Diseases, Tokyo, Japan; 8 Department of Molecular Virology, Tokyo Medical and Dental University, Tokyo, Japan; 9 Artificial Intelligence Center for Health and Biomedical Research, National Institutes of Biomedical Innovation, Health and Nutrition, Osaka, Japan; 10 RIKEN Center for Integrative Medical Sciences, Yokohama, Japan; 11 Institute for Protein Research, Osaka University, Osaka, Japan

**Keywords:** flavivirus replication, VCP/p97, stress granules, NPL4, NS4B, CM, convoluted membrane, DENV, dengue virus, ER, endoplasmic reticulum, ERAD, ER-associated degradation, HCV, hepatitis C virus, JEV, Japanese encephalitis virus, PEI, polyethylenimine, PKR, protein kinase R, SG, stress granule, VP, vesicle packet, WNV, West Nile virus, Y2H, yeast two-hybrid, ZIKV, Zika virus

## Abstract

Flaviviruses are human pathogens that can cause severe diseases, such as dengue fever and Japanese encephalitis, which can lead to death. Valosin-containing protein (VCP)/p97, a cellular ATPase associated with diverse cellular activities (AAA-ATPase), is reported to have multiple roles in flavivirus replication. Nevertheless, the importance of each role still has not been addressed. In this study, the functions of 17 VCP mutants that are reportedly unable to interact with the VCP cofactors were validated using the short-interfering RNA rescue experiments. Our findings of this study suggested that VCP exerts its functions in replication of the Japanese encephalitis virus by interacting with the VCP cofactor nuclear protein localization 4 (NPL4). We show that the depletion of NPL4 impaired the early stage of viral genome replication. In addition, we demonstrate that the direct interaction between NPL4 and viral nonstructural protein (NS4B) is critical for the translocation of NS4B to the sites of viral replication. Finally, we found that Japanese encephalitis virus and dengue virus promoted stress granule formation only in VCP inhibitor-treated cells and the expression of NS4B or VCP attenuated stress granule formation mediated by protein kinase R, which is generally known to be activated by type I interferon and viral genome RNA. These results suggest that the NS4B-mediated recruitment of VCP to the virus replication site inhibits cellular stress responses and consequently facilitates viral protein synthesis in the flavivirus-infected cells.

The genome of the genus Flavivirus is characterized by a single-stranded positive-sense RNA. In humans, flaviviral infections are associated with high rates of morbidity and mortality. Flaviviruses, including dengue virus (DENV), Japanese encephalitis virus (JEV), Zika virus (ZIKV), and West Nile virus (WNV), circulate between arthropod and vertebrate hosts and are etiological factors for major epidemics worldwide. Currently, effective antiflaviviral drugs are not available for human use. Hence, there is an urgent need to develop novel antiflaviviral therapeutic agents (1).

The RNA genome (size: 11 kb) of flaviviruses encodes a large polyprotein, which is translated at the rough endoplasmic reticulum (ER) and integrated into the ER membrane. Subsequently, the polyprotein is processed into three structural proteins (capsid, prM, and E) and seven nonstructural proteins (NS1, NS2A, NS2B, NS3, NS4A, NS4B, and NS5) by viral and cellular proteases (2). NS2A, NS4A, and NS4B, which are essential for virus replication, are not reported to exhibit enzymatic activities. NS2A is thought to function in virion assembly (3, 4), whereas NS4A is known to function in the formation of convoluted membranes at replication organelles (5). The role of NS4B in the viral replication cycle remains unclear. However, NS4B is detected at the vesicle packet (VP) in the replication organelle, which indicates that NS4B functions as a scaffolding protein in viral replication (6, 7). NS4B interacts with NS4A and NS3, a hybrid protein with viral helicase and protease domains. This indicates that the major function of NS4B is to promote the recruitment of NS4A and NS3 (8, 9). Furthermore, WNV NS4B (along with other NS proteins) suppresses experimentally induced stress granule (SG) formation (9, 10).

Valosin-containing protein/p97 (VCP), a hexameric ATPase, regulates diverse cellular functions, including ER-associated degradation (ERAD), autophagy, chromatin remodeling, and DNA repair. VCP carries out its regulatory functions by either extracting ubiquitylated proteins from membranes or cellular structures or by separating them from binding proteins (11). In addition, mutations in VCP result in inclusion body myopathy with Paget disease of the bone and frontotemporal dementia and amyotrophic lateral sclerosis (12). Furthermore, various cofactors, including NPL4, UFD1, p47, p37, and UBXD1, bind to the N-terminal domain of VCP through specific interaction domains or motifs and consequently modulate the VCP-mediated processes (11). Previously, we had reported that siRNA-mediated knockdown of *VCP* markedly downregulated the production of JEV or DENV particles (>10^6^-fold titer reductions) without exerting cytotoxic effects (13). This indicates that VCP is a potential therapeutic target for flaviviral infections.

VCP is involved in at least two steps (capsid uncoating step immediately after virion entry and viral RNA genome replication) of flavivirus replication (14). During capsid uncoating, the VCP complex promotes the transportation of ubiquitinated viral capsids to the proteasome, which leads to the release of the RNA genome into the cytoplasm (15). In viral genome RNA replication, the VCP-mediated ERAD pathway is involved in the degradation of ubiquitinated NS proteins, which is important to maintain the homeostatic levels of viral proteins for efficient flaviviral genome replication (13). However, the mechanisms underlying the antiviral effect of VCP depletion have not been elucidated. In addition to viral capsid uncoating and viral genome replication, VCP may have other roles in flavivirus replication.

Type I interferons and cytoplasmic pattern recognition receptors are involved in typical host antiviral responses. The cellular stress responses, which are considered important antiviral strategies, mainly involve promoting the rapid repression of cellular translation of proteins essential for cell survival. Several viruses repress the cellular translation to facilitate their replication. Hence, this process is a potential therapeutic target for viral diseases (16).

SGs are aggregates of nontranslating messenger ribonucleoprotein complexes formed in response to various environmental and cellular signals (17). SG formation and translational arrest are mediated by the phosphorylation of the α subunit of eukaryotic translation initiation factor 2 (eIF2α), which is catalyzed by several cytoplasmic kinases, including protein kinase R (PKR). PKR, which senses the presence of double-stranded RNA (dsRNA), is important for the response to viral infections (18). Several independent groups have reported that flaviviruses have multiple machineries to inhibit the formation of SG assembly (19, 20, 21, 22, 23). However, the mechanisms involved in the flavivirus-induced SG disassembly are unclear. The aggregates of messenger ribonucleoproteins dissociate after stress release. VCP is reported to play a critical role in SG clearance (24, 25). In this study, NPL4, a cofactor of VCP, was identified as a novel flavivirus host factor. In addition, the potential roles of NPL4 in releasing the viral translational machinery from SGs were determined.

## Results

### NPL4 is a potential VCP cofactor involved in flavivirus propagation

**Figure 1:**
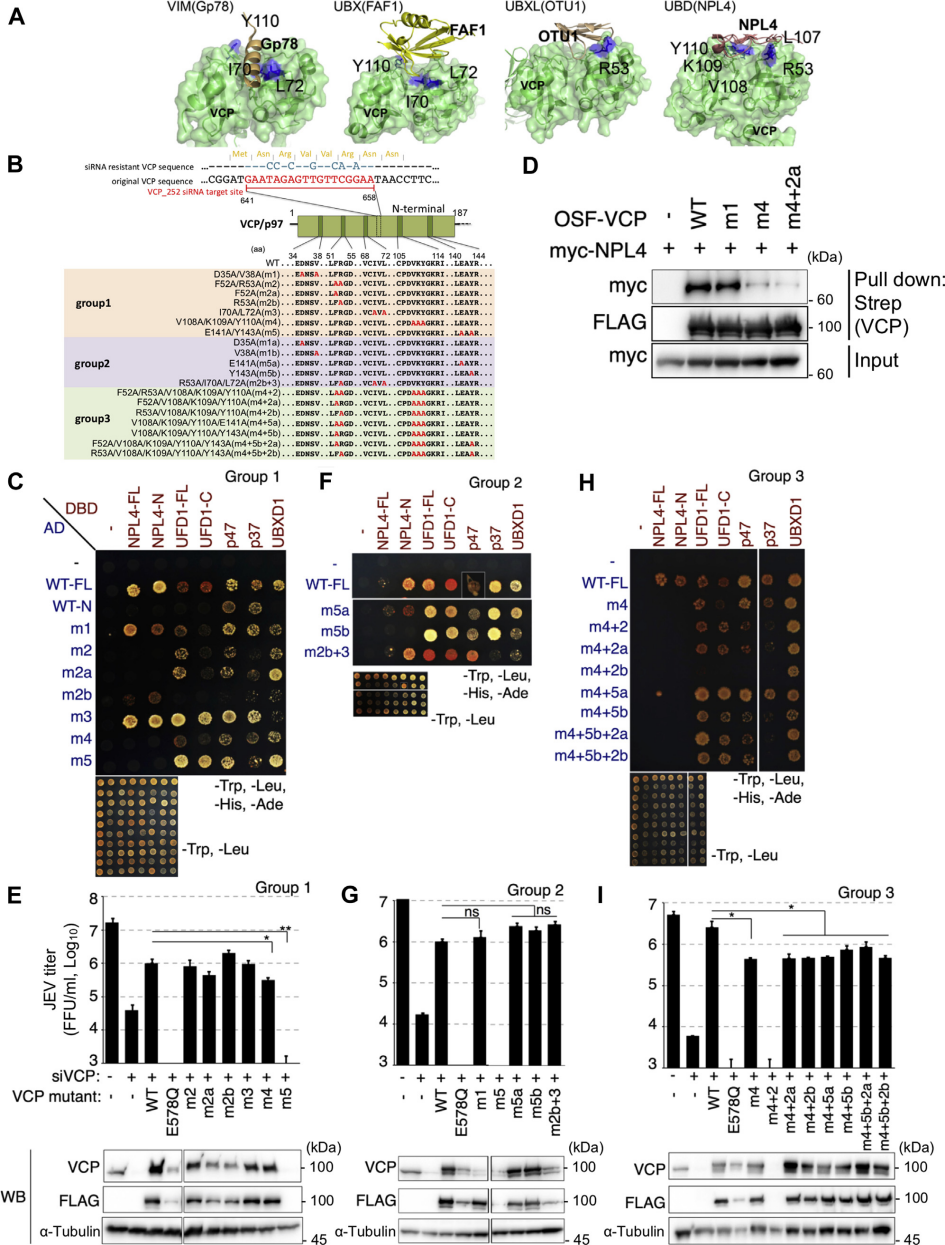
**VCP exerts its function in flavivirus propagation by interacting with NPL4.***A*, structure of the VCP N-terminal domain (*green*) and the binding site for the UBD domain in NPL4 (PDB ID:2PJH, *brown*). Predicted amino acids involved in NPL4 binding are highlighted in *blue*. *B*, schematic structure of the N-terminal domain of VCP (*top*) and the amino acid sequences of wildtype (WT) and mutants (*bottom*). Amino acids labeled in *red* were substituted with alanine. *C*, yeast two-hybrid (Y2H) assay for determining the interactions of VCP mutants of group 1 with cofactors. Yeasts were cotransformed with plasmids expressing AD (activation domain) and DBD (DNA-binding domain) fusion proteins and plated on the control medium (Leu/Trp deficient) or selective medium (Leu/Trp/Ade/His deficient). *D*, pull-down assay of NPL4 with WT or various mutant of VCP. The 293T cells were cotransfected with OSF-tagged VCPs and Myc-tagged NPL4. Bait and prey proteins in the bound fraction were detected using the anti-Myc (*top panel*) or anti-FLAG antibodies (*middle panel*). Prey proteins in the input lysate (Input) fraction were detected using the anti-Myc antibodies (*bottom panel*). *E*, phenotypic rescue using VCP mutants of group 1. The 293A cells were transfected twice with siRNA and rescue vectors. Post second transfection (24 h), the cells were infected with Japanese encephalitis virus (JEV) (multiplicity of infection [MOI] = 0.3). Post infection (48 h), the infectious virus titer in the culture supernatant was measured using a focus forming assay (*top panel*). The E578Q mutant, which lacks in ATPase activity, was used as a control. The protein expression levels were measured using Western blotting with anti-VCP (*second panel*), anti-FLAG (*third panel*), or anti-α-tubulin (*bottom panel*) antibodies. ∗∗*p* < 0.01 and ∗*p* < 0.05 (Student’s *t* test). *F*, Y2H assay for determining the interactions between VCP mutants of group 2 with cofactors. This experimental method was the same as that in *C*. *G*, phenotypic rescue using VCP mutants of group 2. This experimental method was the same as that in *E*. *H*, Y2H assay for determining the interactions of VCP mutants of group 3 with cofactors. This experimental method was the same as that in *C*. *I*, phenotypic rescue using VCP mutants of group 3. This experimental method was the same as that in *E*. ∗*p* < 0.05; ns, not significant (Student’s *t* test). VCP, valosin-containing protein.

To determine the cofactors of VCP critical for JEV propagation, 17 VCP-expressing constructs with a mutation in the cofactor-binding region of the N-terminal domain (1–187 amino acids [aa]) were generated (Fig. 1*A*). These mutants were classified as groups 1, 2, and 3 (Fig. 1*B*). Group 1 comprised five prototype mutants (m1 [D35A/V38A], m2 [F52A/R53A], m3 [I70A/L72A], m4 [V108A/K109A/Y110A], and m5 [E141A/Y143A]) and two subprototype mutants (m2a [F52A] and m2b [R53A]). First, we tested the ability of those mutants to bind with known representative cofactors and its truncation fragments, NPL4-Full length (FL), NPL4-N terminal fragment (N), UFD1-FL, UFD1-C terminal fragment (C), p47-FL, p37-FL, and UBXD1-FL, and we reproduced their bindings to VCP-N terminal region using a yeast two-hybrid (Y2H) assay (Fig. 1*C*). All tested cofactors interacted with the full-length VCP protein (Fig. 1*C*, row 2). Among these, only p47 and p37 interacted with the N-terminal fragment of VCP (Fig. 1*C*, row 3). The VCP mutants m1 and m3 also interacted with all tested cofactors. However, the m2, m2a, m4, and m5 mutants did not interact with NPL4 alone, whereas m2b did not interact with UFD1 alone. The inability of the VCP m4 mutant to bind to NPL4 was verified using a pull-down assay with the epitope-tagged proteins expressed in 293T cells (Fig. 1*D*).

Next, the ability of these mutants to rescue impaired JEV propagation in VCP-depleted cells was examined (Fig. 1*E*). The E578Q mutant, which lacks in ATPase activity, was used as a control. Only m4 mutants could not effectively rescue VCP-mediated viral propagation (the rescue rate was only 31% of that of the wildtype [WT] control). The m5 mutant also did not rescue VCP-mediated viral propagation function. However, we could not determine whether this mutant is functionally null because its expression was not detected in cells. The viral propagation rescue rates of the m2b mutants, which could not bind to UFD1 alone, were similar to those of WT VCP. This suggests that the binding of UFD1 is dispensable for VCP function in viral propagation.

Based on the results of experiments with group 1 mutants, this study focused on m5, m2b, and m3 mutations. The m5 (E141A/Y143A) mutation was split into m5a (E141A) and m5b (Y143A) mutations. The results of the Y2H assay revealed that the m5a mutant interacted with all tested cofactors, whereas the m5b mutant did not interact with NPL4 alone (Fig. 1*F*). In addition, a combinatorial mutant with m2b (R53A) and m3 (I70A/L72A) mutations was generated and named m2b + 3 (R53A/I70A/L72A). This mutant exhibited significantly decreased binding to p37. These three mutants were expressed at reasonable levels and markedly rescued virus propagation in VCP-depleted cells (Fig. 1*G*). This indicates that the binding of VCP to p37 and both m5a and m5b mutation sites (E141 and Y143) are not important for VCP-mediated virus propagation (Fig. 1*G*). In contrast to the m2b mutant, the m2b+3 mutant interacted with UFD1 (Fig. 1, *C* and *F*). One explanation for this observation is additional mutations in m2b may have induced a conformational change in the N-terminal region of VCP and consequently restored its ability to bind to UFD1.

Next, the following seven additional combinatorial mutants with m4 were generated: m4+2 (F52A/R53A/V108A/K109A/Y110A), m4+2a (F52A/V108A/K109A/Y110A), m4+2b (R53A/V108A/K109A/Y110A), m4+5a (V108A/K109A/Y110A/E141A), m4+5b (V108A/K109A/Y110A/Y143A), m4+5b+2a (F52A/V108A/K109A/Y110A/Y143A), and m4+5b+2b (R53A/V108A/K109A/Y110A/Y143A). The binding of these mutants to cofactors and their ability to rescue virus propagation in VCP-depleted cells were examined (Fig. 1, *H* and *I*). All additional m4 mutants exhibited impaired binding to NPL4, whereas the m4+2b mutant exhibited decreased binding to UFD1, p47, and p37. Moreover, all these mutants could not effectively rescue virus propagation in VCP-depleted cells. The rescue rates of all additional m4 mutants were similar to those of the parental m4 mutant, which exhibited 18% to 34% rescue rates of those of the WT control. None of the additional m4 mutants exhibited additive or synergistic inhibitory effects. These findings indicated that only the m4 mutation sites, namely, V108, K109, and Y110, were critical for VCP-mediated virus propagation. The other sites that were tested for their effects on virus propagation were dispensable for VCP-mediated virus propagation. The m4 mutant did not interact with NPL4 alone but interacted with other cofactors. This suggests that VCP binding to NPL4 is critical for VCP-mediated viral propagation and that VCP binding to other cofactors is dispensable for this function.

### Flavivirus NS4B interacts with NPL4–VCP complex

**Figure 2:**
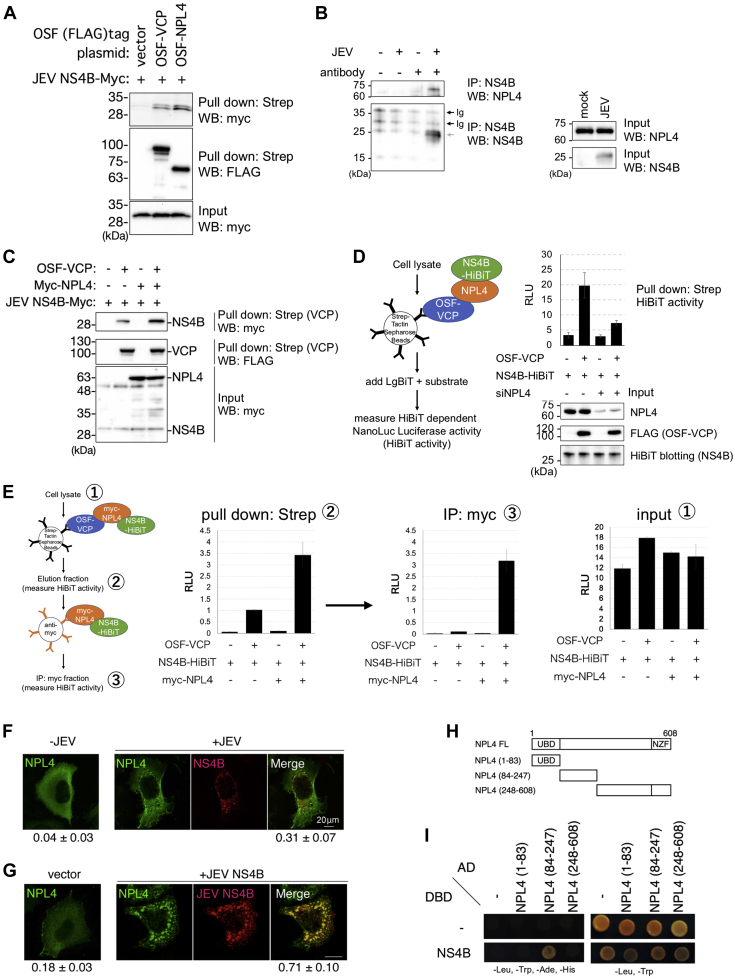
**NPL4-VCP complex interacts with Japanese encephalitis virus (JEV)-NS4B.***A*, pull-down assay of JEV-NS4B with VCP and NPL4. The 293T cells were cotransfected with OSF-VCP or OSF-NPL4 with Myc-JEV-NS4B. Bait and prey proteins in the bound fraction were detected using the anti-Myc (*top panel*) or anti-FLAG antibodies (*middle panel*). Prey proteins in the lysate (Input) were detected (*bottom panel*). *B*, immunoprecipitation of JEV-NS4B with endogenous NPL4 using 293T cells infected with JEV (MOI = 1.0) or mock for 48 h. Bait and prey proteins bound to anti-JEV-NS4B or control antibody–conjugated beads detected using the anti-NPL4 (*left top panel*) or anti-JEV-NS4B (*left bottom panel*) antibodies. *Black* and *gray arrows* indicate immunoglobulin bands and NS4B, respectively. Prey proteins in the input lysate (Input) fraction were detected using the anti-NPL4 (*right top panel*) or anti-JEV-NS4B (*right bottom panel*) antibodies. *C*, NPL4 expression promotes the interaction between VCP and NS4B. The 293T cells were cotransfected with OSF-tagged VCPs and Myc-tagged JEV-NS4B with or without Myc-NPL4. Bait and prey proteins in the bound fraction were detected using the anti-Myc (*top panel*) or anti-FLAG (*middle panel*) antibodies. Prey proteins in the input lysate (Input) fraction were detected using the anti-Myc antibodies (*bottom panel*). *D*, effect of NPL4 knockdown on the interaction between VCP and NS4B. Schematic presentation of pull-down HiBiT assay is shown in the *left panel*. 293T cells were transfected twice with siRNA, followed by the OSF-VCP and NS4B-HiBiT expression plasmids. After cell lysis and pull-down with Strep-Tactin Sepharose beads, HiBiT-dependent NanoLuc luciferase activity (HiBiT activity) in the bead-bound fraction was measured (graph). Prey proteins in the input lysate were detected using the anti-NPL4 (*top panel*) antibody, anti-FLAG (*middle panel*) antibody, or HiBiT blotting (*bottom panel*). *E*, two-successive pull-down experiments for NS4B–NPL4–VCP ternary complex. Schematic presentation of two-successive pull-down HiBiT assays is shown in the *left panel*. 293T cells were transfected with NS4B-HiBiT, with or without Myc-NPL4 and OSF-VCP. After cell lysis and pull-down with the Strep-Tactin Sepharose beads, HiBiT activity was measured in the bead-bound fractions (*second panel*). After eluting bead-bound proteins by adding destiobiotin, the eluted fractions were mixed with anti-Myc antibody–conjugated Sepharose beads. HiBiT activities were then measured in the bead-bound fractions (*third panel*). HiBiT activities in the input fractions were measured (*right panel*). *F* and *G*, colocalization of NPL4 and JEV-NS4B in JEV-infected cells or cotransfected cells. Post infection (24 h), JEV (MOI = 1.0)-infected HeLa cells were transfected with OSF-NPL4 and fixed. The levels of OSF-NPL4 and NS4B were detected with the anti-FLAG (*green*) or anti-JEV-NS4B (*red*) antibodies (*F*). HeLa cells were cotransfected with OSF-NPL4 (*green*) and JEV-NS4B-Myc (*red*) (*G*). The numbers under each image indicate Mander’s colocalization coefficient R. The scale bar represents 20 μm (*F* and *G*). *H*, schematic structure of NPL4 and its fragments used in (*I*). UBD, “ubiquitin-like” domain; NZF, Npl4 zinc finger or RanBP2/Nup358 zinc finger. *I*, yeast two-hybrid assay for examining the interaction between NPL4 and JEV-NS4B fragments (2393–2447 aa). Each pair of plasmids includes one plasmid that expresses AD fused to the fragments of NPL4 and one plasmid that expresses DBD fused to fragments derived from JEV polypeptide containing the cytoplasmic region. Plasmids were transformed into the yeast strain AH109. Cells were plated on the control medium (*right panel*, Trp^−^ and Leu^−^) or the selective medium (*left panel*, Trp^−^, Leu^−^, His^−^, and Ade^−^). The numbers in each column indicate the amino acid regions expressed in the yeast cells. VCP, valosin-containing protein.

Previously, proteome analysis revealed that both NPL4 and VCP coprecipitated with JEV-NS4B and DENV-NS4B (13). Hence, these interactions were confirmed using the reciprocal pull-down assay. As shown in Figure 2*A*, JEV-NS4B coprecipitated with both VCP and NPL4. These interactions were confirmed *via* the immunoprecipitation assay using antibodies directly bound to JEV-NS4B and endogenous NPL4. Endogenous NPL4 was coprecipitated with NS4B using a JEV infection–dependent method, indicating the specific interaction between NS4B and NPL4 (Fig. 2*B*). NPL4 is a well-known cofactor involved in the substrate recognition of VCP. Hence, the effect of NPL4 expression on the interaction between NS4B and VCP was evaluated. NPL4 overexpression was promoted, whereas NPL4 knockdown impaired the interaction between VCP and NS4B (Fig. 2, *C* and *D*), suggesting that NPL4 may act as a bridge for the interaction between VCP and NS4B. Two successive pull-down experiments using Strep-tag and Myc-tag affinity beads showed that NPL4 has a central role in the interaction between the NS4B and NPL4-VCP complexes (Fig. 2*E*). These data suggest that NPL4 functions as a cofactor of VCP and mediates the interaction between VCP and NS4B during viral infection.

Furthermore, NPL4 colocalized with NS4B. NPL4 diffusely localized at the cytoplasm when it was expressed alone but translocated to the NS4B-positive compartment in JEV-infected cells or NS4B-transfected cells (Fig. 2, *F* and *G*). This further indicated that NPL4 interacts with NS4B. The direct interaction between NPL4 and NS4B was examined using the Y2H assay (Fig. 2, *H* and *I*), which is suitable for analyzing the cytosolic proteins. Hence, the interaction of all possible cytosolic fragments of JEV-NS4B with NPL4 was examined. Strong signals are observed with small proteins. Therefore, NPL4 was divided into three fragments (1–83, 84–247, and 248–608 aa) and the interactions of these fragments with the NS4B fragments were examined. The combination of DNA-binding domain fused to NS4B (2393–2447 aa) and activation domain fused to NPL4 (84–247 aa) yielded a positive signal, indicating that these peptides directly interacted with each other (Fig. 2*I*). These results suggested that NPL4 directly interacted with the NS4B cytoplasmic loop.

### NPL4–VCP complex is required at early stage in viral life cycle

**Figure 3:**
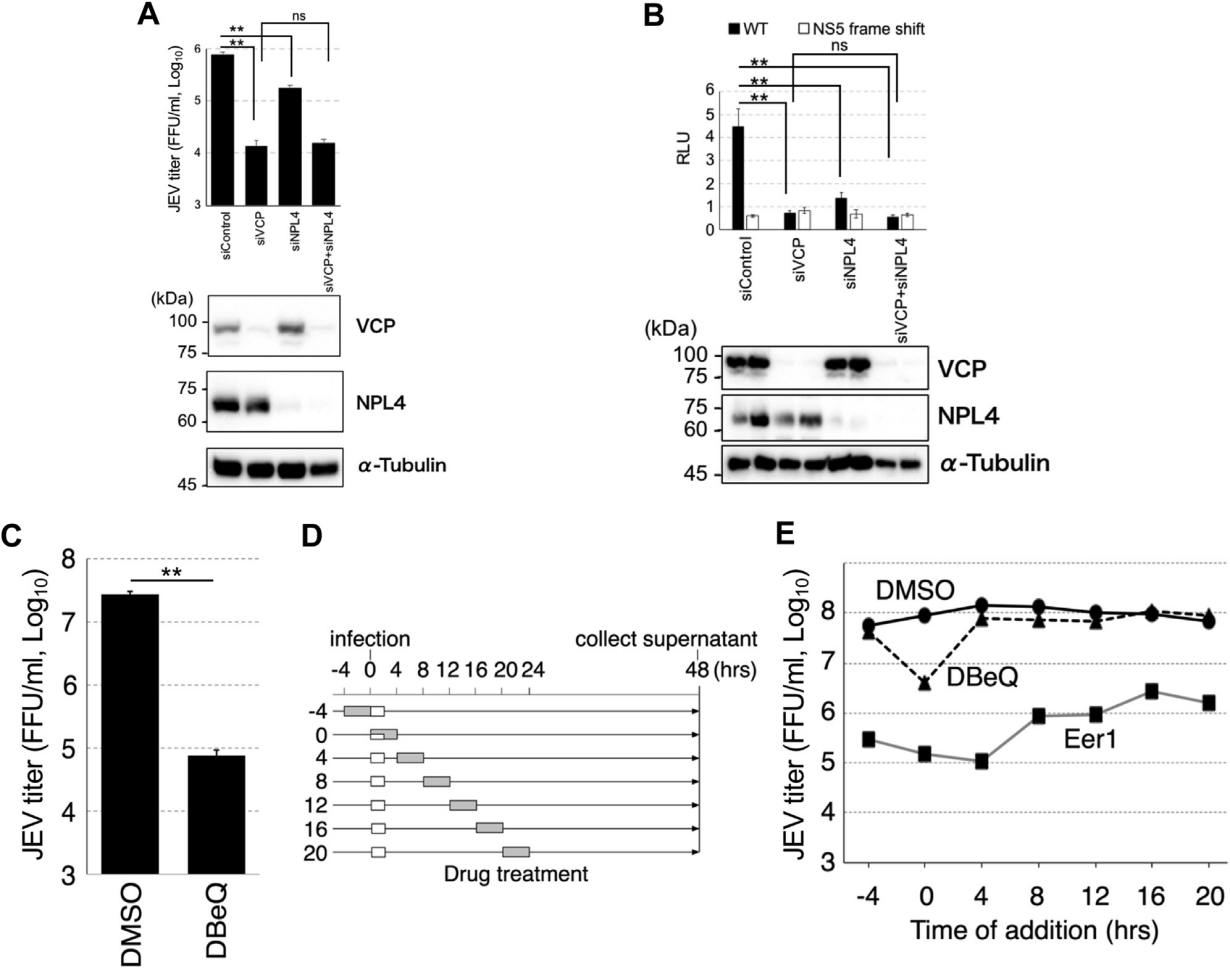
**VCP ATPase is important for early stage of viral propagation**. *A*, effect of *NPL4* and/or *VCP* knockdown on JEV propagation. 293T cells were transfected with siRNA against NPL4 and/or VCP twice for 24 h. The cells were then infected with JEV (MOI = 0.3). Virus titers in the supernatant 48 h post infection were measured (*top graph*). ∗∗*p* < 0.0001; ns, not significant (Tukey's multiple comparison test). The levels of VCP (*second panel*), NPL4 (*third panel*), and α-tubulin (*bottom panel*) were determined using Western blotting. *B*, effect of *NPL4* and/or *VCP* knockdown on Dengue virus (DENV) genome replication. 293T cells were transfected with siRNA against NPL4 and/or VCP twice for 24 h, followed by transfection with NanoLuc expressing the DENV subgenomic replicon RNA-expressing plasmid. NanoLuc-dependent luciferase activities were measured in the cell lysate at 72 h post transfection (*top graph*). ∗∗*p* < 0.001; ns, not significant (Tukey's multiple comparison test). The levels of VCP (*second panel*), NPL4 (*third panel*), and α-tubulin (*bottom panel*) were determined using Western blotting. *C*, effect of DBeQ on JEV propagation. The 293A cells were infected with JEV (MOI = 0.3) for 2 h and treated with 10 μM of DBeQ for 4 h. Post infection (48 h), the infectious virus titer in the culture supernatant was measured using a focus forming assay. ∗∗*p* < 0.01 (Student’s *t* test). *D* and *E*, time of addition assay. *D*, schematic diagram of DBeQ treatment. The 293A cells were infected with or without JEV (MOI = 0.3) and treated with 10 μM of DBeQ or 10 μM of Eer1 for 4 h at the indicated time points. Virus inoculation (*white box*) and drug treatment (*gray box*) time frames are indicated. *E*, results of time of the addition assay. All supernatants were collected 48 h post infection to determine the JEV infectious titer using a focus forming assay. JEV, Japanese encephalitis virus; MOI, multiplicity of infection; VCP, valosin-containing protein.

To examine the role of NPL4 in flavivirus propagation, the effect of *NPL4* knockdown on viral propagation was examined. Treatment with siRNAs efficiently depleted the endogenous NPL4 levels and significantly impaired JEV propagation (Fig. 3*A*). Two different targeted siRNAs were evaluated, and similar results were obtained (data not shown). Treatment with NPL4 siRNAs also significantly impaired DENV replication in the subgenomic replicon cells (Fig. 3*B*). These findings indicate that NPL4 is required for flavivirus replication. Furthermore, the simultaneous knockdown of *NPL4* with *VCP* did not demonstrate any additive or synergistic inhibitory effect on either JEV propagation or DENV replication (Fig. 3, *A* and *B*), suggesting that NPL4 and VCP play roles in the same pathway.

A recent study has reported that the ATPase activity of VCP is required for flavivirus propagation (13). Hence, the cells infected with the virus for 2 h were treated with specific small-molecule VCP ATPase inhibitors, such as DBeQ (26), for 4 h. Treatment with the VCP ATPase inhibitors impaired the production of infectious JEV in the supernatant (Fig. 3*C*), which was consistent with the results of a previous study (13). A 4-h pulse treatment with DBeQ just after virus inoculation was sufficient to reduce the viral titers. However, the inhibitory effect of DBeQ on virus production was not observed at other time points (Fig. 3, *D* and *E*). These results suggest that VCP functions in the early and not the late stage of viral infection. Treatment with the irreversible ERAD inhibitor eeyarestatin I (EerI) (27) impaired virus production irrespective of the timing of inhibitor treatment (Fig. 3, *D* and *E*).

### Virus infection induces SG formation in the absence of NPL4–VCP function

**Figure 4:**
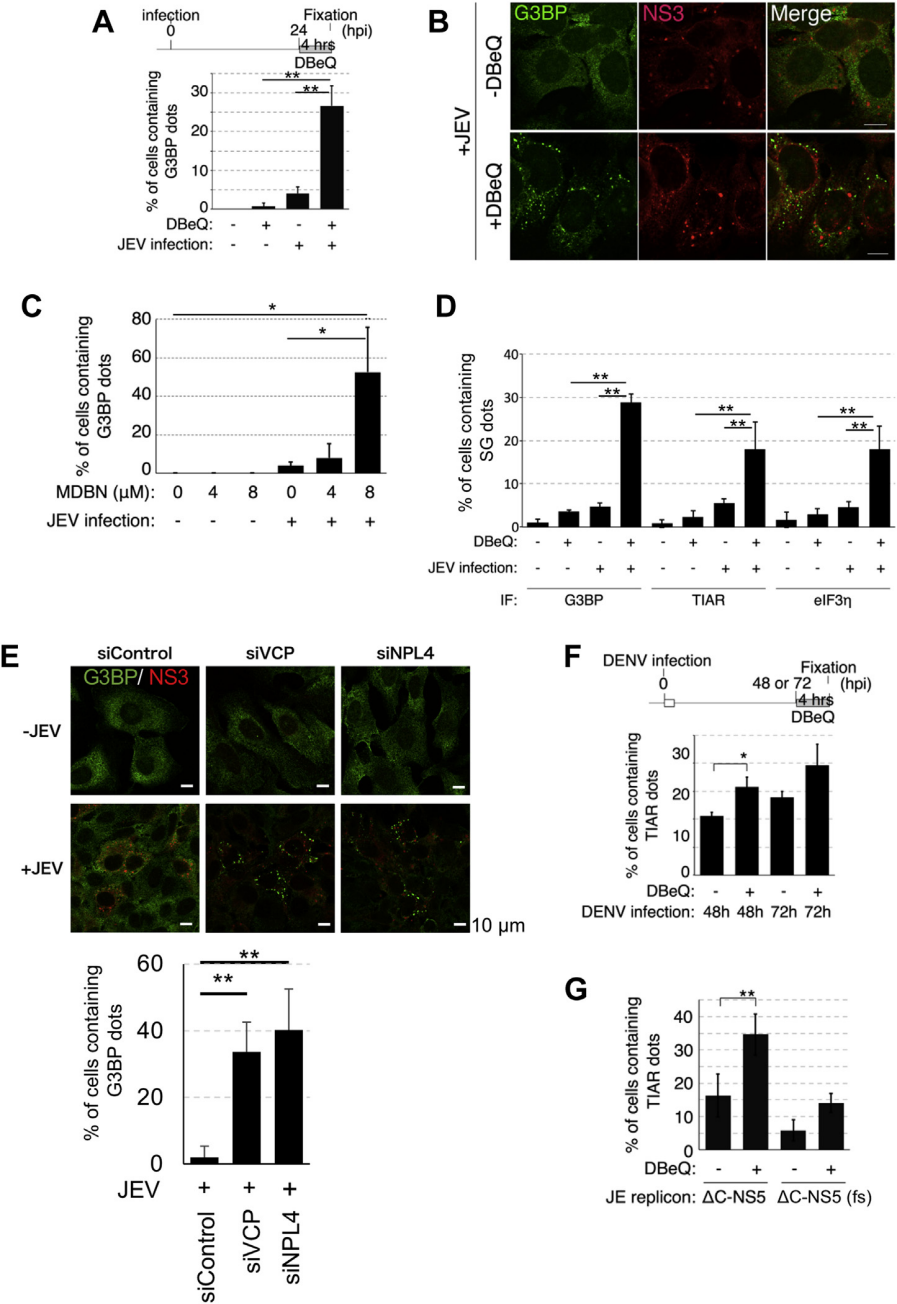
**VCP inhibition induces stress granule (SG) formation in flavivirus-infected cells.***A* and *B*, effect of DBeQ treatment and/or JEV infection on SG formation. Schematic diagram illustrating DBeQ treatment (*A*, *upper*). The number of cells containing SGs was counted (*A*, *lower*), after staining the cells with anti-G3BP (*green*) and JEV-NS3 (*red*) antibodies (*B*). ∗∗*p* < 0.01 (Student’s *t* test). The scale bar represents 20 μm. *C*, 3,4-Methylenedioxy-b-nitrostyrene (MDBN: VCP inhibitor) dose dependently increased the formation of SGs in JEV-infected cells. Huh7 cells were treated with MDBN at the indicated amount for 4 h post infection (24 h). The number of cells containing SGs was counted, after staining the cells with the anti-G3BP antibodies. ∗*p* < 0.05 (Student’s *t* test). *D*, effect of DBeQ treatment and/or JEV infection on SG formation. Huh7 cells were mock infected or infected with JEV (MOI = 1.0) and treated with or without DBeQ (5 μM) for 4 h post infection (24 h). The number of cells containing SGs was counted, after staining the cells with anti-G3BP, anti-TIAR, or anti-eIF3η antibodies. ∗∗*p* < 0.01 (Student’s *t* test). *E*, effect of VCP or NPL4 knockdown on JEV infection–mediated SG formation. Huh7 cells were transfected twice with siRNA for a 24-h interval, followed by JEV or mock-infected cells (MOI = 1.0) for 24 h. The cells were fixed and stained with anti-G3BP (*green*) and anti-JEV-NS3 (*red*) antibodies. The scale bar represents 10 μm. The numbers of cells containing G3BP dots among the NS3-positive cell population were plotted in the *bottom graph*. ∗∗*p* < 0.05 (Student’s *t* test). *F*, effect of DBeQ treatment and/or DENV infection on SG formation. Schematic diagram of DBeQ treatment (*upper*). Huh7 cells were subjected to mock infection or DENV infection (MOI = 1.0) at 0 h and treated with or without 5 μM of DBeQ for 4 h post infection (48 or 72 h). The number of cells containing SGs was counted, after staining the cells with the anti-TIAR antibodies. ∗*p* < 0.05 (Student’s *t* test). *G*, SG formation in DBeQ-treated subgenomic replicon cells. SG puncta in the cells transfected with JE replicon, a plasmid expressing JEV subgenomic-replicon RNA, were detected using the anti-TIAR antibodies. A JE replicon [C-NS5(fs)] with a frame-shift mutation (fs) within the NS5 coding region was used as a replication-incompetent construct. ∗∗*p* < 0.01 (Student’s *t* test). JEV, Japanese encephalitis virus; MOI, multiplicity of infection; VCP, valosin-containing protein.

Buchan *et al.* (24) reported that VCP is involved in dissociating SGs after cellular stresses are relieved. Thus, the effect of VCP inhibitors on SG formation was examined in virus-infected cells. The number of cells with G3BP-positive SGs in the DBeQ-treated and JEV-infected group was significantly higher than that in the DBeQ-treated and mock-infected group (Fig. 4, *A* and *B*). Furthermore, treatment with 3,4-methylenedioxy-b-nitrostyrene (MDBN; a VCP inhibitor) dose dependently and significantly increased the number of cells with SGs in the JEV-infected cells (Fig. 4*C*). These results suggest that these are not the specific phenotypes observed with DBeQ treatment. To examine the VCP inhibition–mediated SG formation, the number of cells with SG formation expressing other SG markers among the virus-infected cells was examined in the same experiments. DBeQ significantly increased the production of TIAR-positive or eIF3η-positive SGs in the virus-infected cells (Fig. 4*D*). This suggested that these SGs are not G3BP-specific structures. Although VCP or NPL4 knockdown reduced the viral genome replication, the basal levels of viral genome replication and viral protein expression were observed in these cells. The number of SGs-positive cells was increased upon VCP or NPL4 depletion in a JEV infection–dependent manner (Fig. 4*E*). SG formation was also observed in DENV-infected cells (Fig. 4*F*). Thus, the induction of SG formation may be a common feature of flaviviruses. VCP inhibition–mediated SG formation was also observed in JEV subgenomic replicon cells (Fig. 4*G*). In the JEV subgenomic replicon cells, viral protein translation, which is accompanied by viral genome replication, proceeds without the viral structural proteins capsid, prM, and E. This suggested that viral structural proteins are not involved in this process. These findings indicate that flavivirus genome replication or related event potentially induces SG formation in the absence of VCP function.

**Figure 5:**
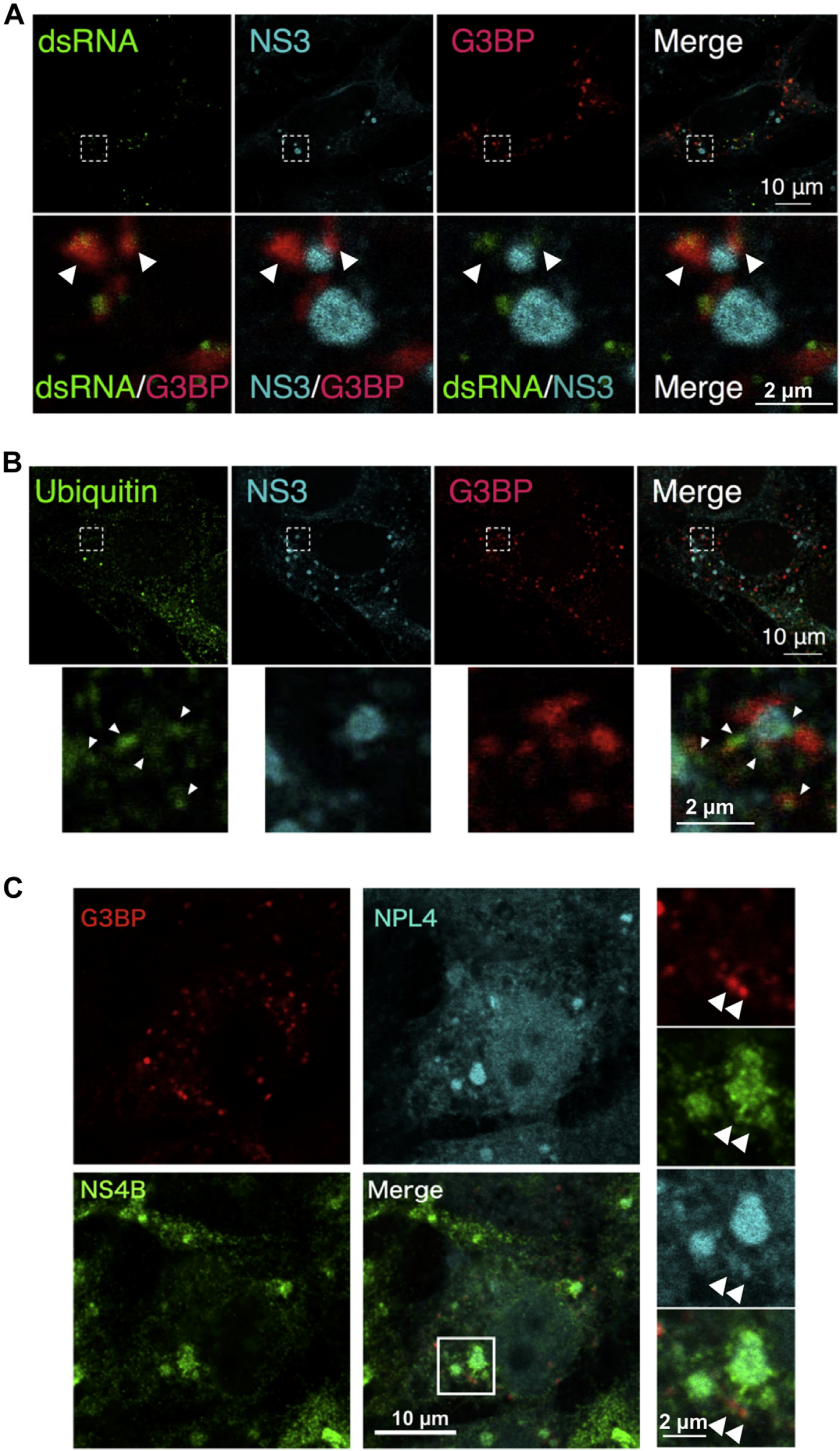
**Stress granules (SGs) localize adjacent to replication organelle.***A*, SGs localized adjacent to the replication organelle. Huh7 cells stably expressing mCherry-G3BP (*red*) were infected with JEV and treated with 5 μM DBeQ for 4 h post infection (24 h). The cells were fixed and stained with anti-double-stranded RNA (dsRNA) (*green*) and anti-JEV-NS3 (*cyan*) antibodies. Arrowheads indicate G3BP-positive SGs. *B*, accumulation of ubiquitinated proteins in the replication organelles. mCherry-G3BP-expressing Huh7 cells were infected with JEV (MOI = 1.0) and treated with 5 μM DBeQ for 4 h post infection (24 h). The cells were fixed and stained with anti-ubiquitin (FK2, *green*) and anti-JEV-NS3 antibodies (*cyan*). The *white arrowheads* indicate ubiquitinated proteins accumulated around the viral replication organelles. The scale bar represents 10 μm. *C*, NPL4 is localized at the replication organelles and SGs. mCherry-tagged G3BP (*red*) and moxCerulean3-tagged NPL4 (*cyan*)–expressing Huh7 cells were infected with JEV (MOI = 1.0) and treated with 10 μM DBeQ for 4 h post infection (48 h). The cells were fixed and stained with the anti-NS4B antibodies (*green*). The scale bar represents 10 μm. JEV, Japanese encephalitis virus; MOI, multiplicity of infection.

Immunofluorescence analysis revealed that the G3BP puncta overlapped with dsRNA. The G3BP signals were adjacent to NS3, which was detected in the convoluted membrane (CM) (Fig. 5*A* arrowhead). A previous study indicated that G3BP was ubiquitinated during its disassembly (25). Ubiquitin is also targeted for NPL4–VCP activity (11). Although the ubiquitin signals overlapped with NS3, VCP inhibition–induced SG was partially colocalized with ubiquitin signals (Fig. 5*B*). This suggests that SGs formed close to the viral replication organelles could be in the disassembly process. Furthermore, partial NPL4 signals overlapped with both NS4B and G3BP (Fig. 5*C*). This indicated that the NPL4–VCP complex is recruited to the replication organelle through interaction with NS4B and that this complex modulates the formation of SG adjacent to the viral replication organelle.

**Figure 6:**
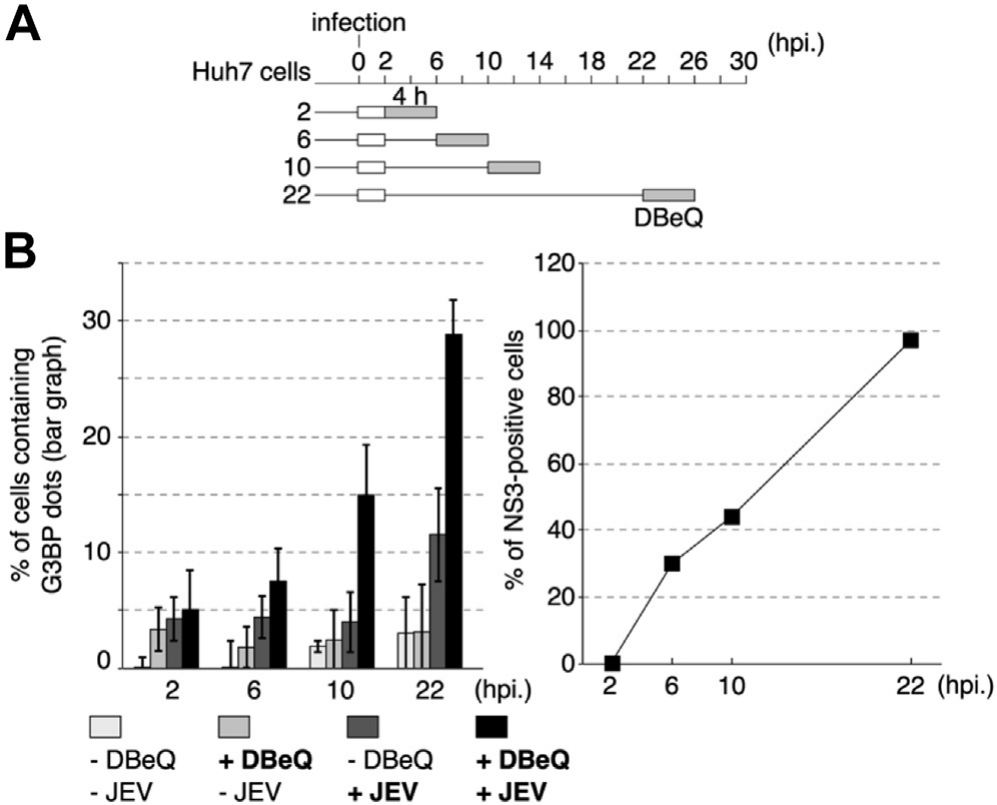
**VCP inhibition–induced stress granule formation is correlated with viral protein expression.***A*, schematic diagram of VCP inhibitor treatment. *Gray* and *white boxes* indicate the time frame of the treatment with DBeQ and virus inoculation, respectively. *B*, JEV or mock-infected (MOI = 1.0) cells were treated with or without DBeQ (5 μM) for 4 h post infection (2, 6, 10, and 22 h). The cells were then fixed and stained with anti-G3BP and anti-NS3 antibodies to detect stress granules and virus infection. The number of stress granule–positive cells (*left panel*) and NS3-positive cells (*right panel*) in the total cell culture was counted. JEV, Japanese encephalitis virus; MOI, multiplicity of infection; VCP, valosin-containing protein.

To further analyze VCP inhibition–mediated SG formation, the correlation between viral protein expression and VCP inhibition was examined in JEV-infected cells. The effect of DBeQ on SG formation at an early stage of infection was examined. As shown in Figure 6, the number of NS3-positive cells gradually increased to almost 100% at 22 h post infection. The number of SGs increased between 6 and 10 h post infection, a time frame during which viral proteins were detected. These results suggest that DBeQ-induced SG formation is related to viral protein expression.

### VCP suppresses SG accumulation in virus-infected cells

**Figure 7:**
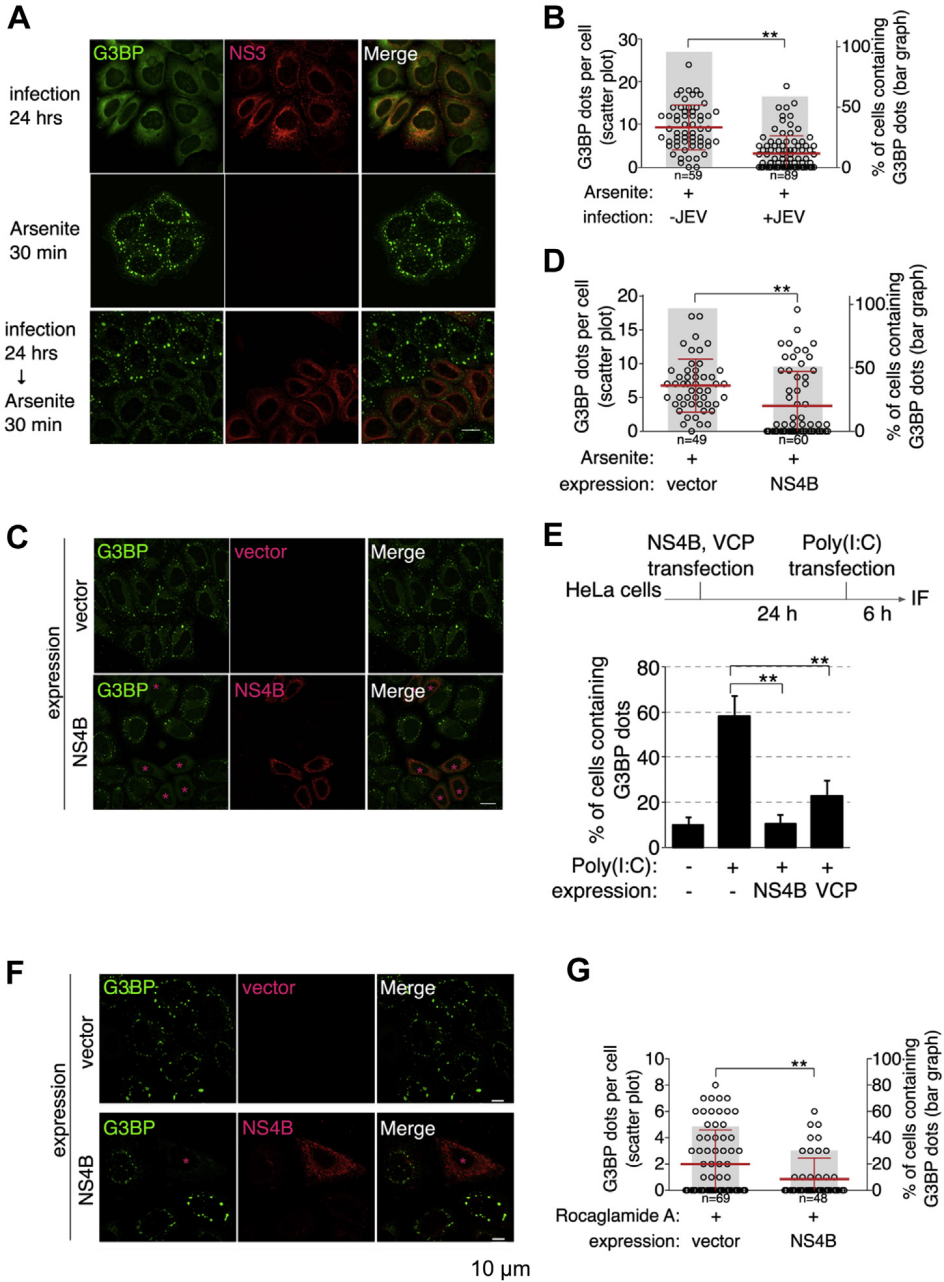
**Expression of NS4B and JEV infection impair stress-induced stress granule formation.***A* and *B*, inhibition of oxidative stress–induced SG formation in JEV-infected cells. HeLa cells were infected with JEV (MOI = 1.0). Post infection (24 h), the cells were treated with 100 μM arsenite for 30 min, fixed, and stained with anti-G3BP (*green*) and anti-JEV-NS3 (*red*) antibodies. The scale bar represents 20 μm (*A*). The number of G3BP puncta in each cell was plotted using a scatter plot. The number of cells with G3BP puncta is shown in the bar graph (*B*). ∗∗*p* < 0.01 (Student’s *t* test). *C* and *D*, inhibition of oxidative stress–induced SG formation in JEV-NS4B-transfected cells. The HeLa cells were transfected with JEV-NS4B-Myc expression plasmid for 24 h. Next, the cells were treated with 100 μM arsenite for 30 min, fixed, and stained with anti-G3BP (*green*) and anti-Myc (*red*) antibodies. *Asterisks* indicate cells exhibiting strong NS4B expression. The scale bar represents 20 μm (*C*). The number of G3BP puncta in each cell was plotted as a scatter plot. The number of cells with G3BP puncta is shown in the bar graph (*D*). ∗∗*p* < 0.01 (Student’s *t* test). *E*, inhibition of poly(I:C)-induced SG formation in JEV-NS4B-transfected cells. The HeLa cells were transfected with JEV-NS4B-Myc or Myc-VCP expression plasmid for 24 h. The cells were then transfected with 100 ng/ml of poly(I:C) for 6 h, fixed, and stained with the anti-G3BP antibodies. The number of cells with G3BP puncta was counted and indicated in the bar graph. ∗∗*p* < 0.01 (Student’s *t* test). *F* and *G*, inhibition of rocaglamide A (Roc-A)-induced SG formation in JEV-NS4B-transfected cells. The HeLa cells were transfected with JEV-NS4B-Myc expression or empty vector for 24 h. The cells were then treated with 500 nM Roc-A for 1 h, fixed, and stained with the anti-G3BP and anti-Myc antibodies. *Asterisk* indicates NS4B-positive cells (*F*). The number of G3BP puncta in each cell was plotted as a scatter plot. The number of cells with G3BP puncta is shown in the bar graph (*G*). ∗∗*p* < 0.01 (Student’s *t* test). JEV, Japanese encephalitis virus; MOI, multiplicity of infection; SG, stress granule; VCP, valosin-containing protein.

Viral infection activates the antiviral stress responses, such as the assembly of RNA granules in the host cells to inhibit mRNA translation. Previous studies have elucidated the mechanisms underlying the virus-mediated modulation of the assembly/disassembly of RNA granules. However, the molecular mechanisms underlying the RNA granule formation are unclear. Recently, two studies have reported that VCP plays an important role in SG clearance (24, 28). Thus, the status of SG formation in virus-infected cells was examined. Consistent with the previous findings in DENV-infected and WNV-infected cells (19, 29), JEV infection significantly impaired oxidative stress–induced SG formation (20). Treatment with arsenite or polyinosinic:polycytidylic (poly (I:C); a mimic of viral dsRNA) promoted SG formation through eIF2α phosphorylation. Thus, the effect of NS4B expression on SG formation through eIF2α phosphorylation was examined. In arsenite-treated cells, virus infection significantly impaired arsenite-induced SG accumulation (Fig. 7, *A* and *B*). Similar results were obtained upon the expression of NS4B. The G3BP puncta were less or not observed in JEV-NS4B–expressing cells (Fig. 7, *C* and *D*). This suggested that NS4B is involved in the inhibition of SG formation in virus-infected cells. In addition, NS4B expression mitigated poly (I:C)-induced SG accumulation (Fig. 7*E*). These results suggest that NS4B inhibits the eIF2α phosphorylation–dependent SG induction pathway. Interesting, VCP expression also mitigated poly (I:C)-induced SG accumulation (Fig. 7*E*), suggesting that the VCP complex is involved in the same pathway. In addition to the eIF2α phosphorylation–dependent pathway, this study examined the effect of NS4B expression on SG formation through the eIF2α phosphorylation–independent pathway. The cells were treated with rocaglamide A, an inhibitor of eIF4A and an SG inducer. NS4B expression impaired SG formation in rocaglamide A–treated cells (Fig. 7, *F* and *G*). These results suggest that NS4B and VCP suppress SG accumulation in the downstream process of SG formation cycle.

**Figure 8:**
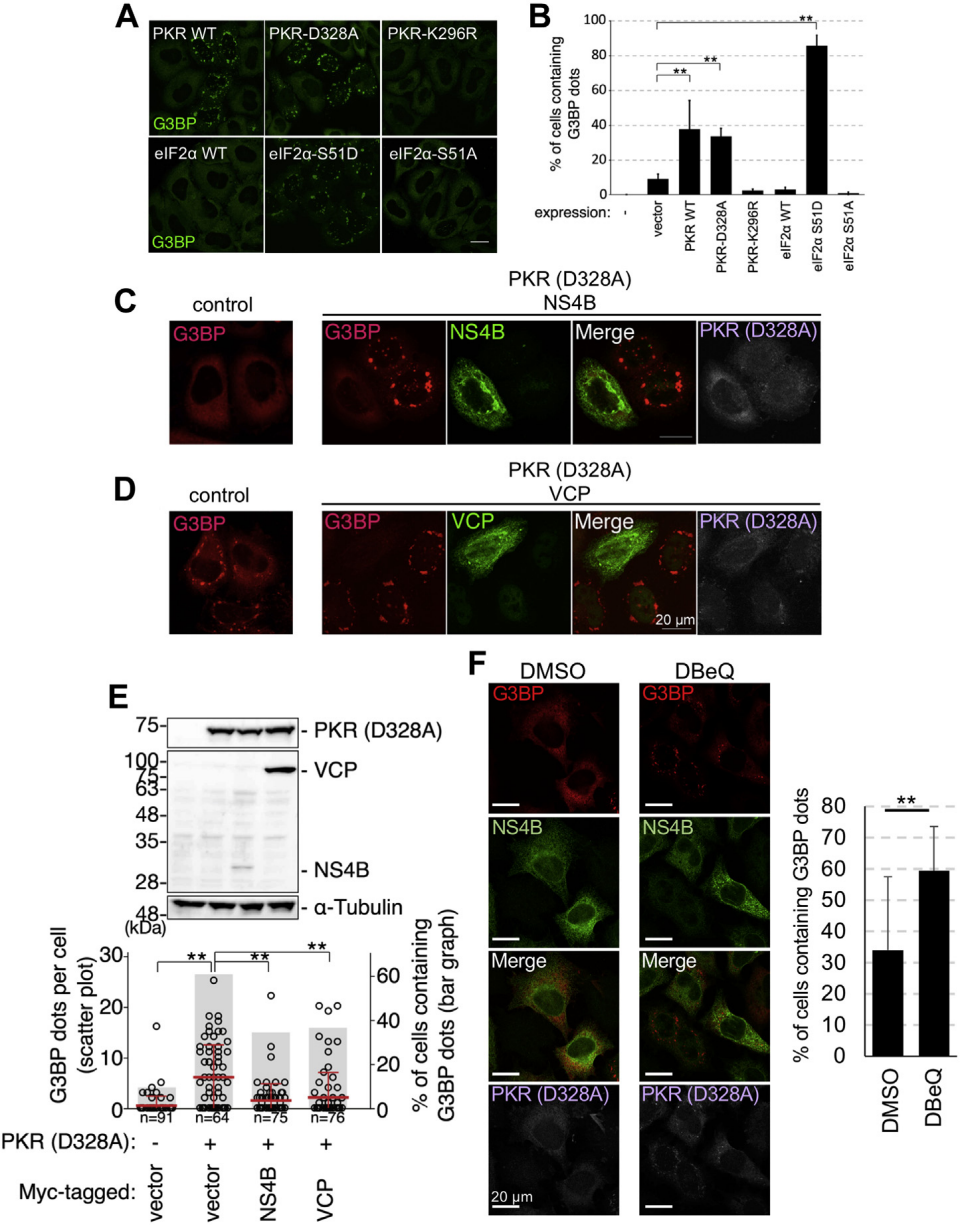
**Expression of NS4B and Japanese encephalitis virus infection impair activated protein kinase R (PKR)-induced SG formation.***A* and *B*, SG induction in cells expressing WT PKR, a constitutively active PKR (PKR-D328A), a kinase dead PKR (PKR-D296R), WT eIF2α, a phosphomimetic form of eIF2α (eIF2α-S51D), or a phosphorylation dead form of eIF2α (eIF2α-S51A). *A*, post transfection (24 h) with PKR-WT, PKR-D328A, PKR-K296R (*A*, *upper*) or eIF2α-WT, eIF2α-S51D, eIF2α-S51A (*A*, *lower*) expression vector, the HeLa cells were fixed and stained with anti-G3BP antibody. The scale bar represents 20 μm. *B*, the number of cells with G3BP puncta was counted. ∗∗*p* < 0.01 (Student’s *t* test). *C* and *D*, NS4B and VCP expression suppresses PKR-induced SG formation. HeLa cells were cotransfected with OSF-PKR-D328A, mCherry-G3BP (*red*), and a plasmid expressing either NS4B-Myc or Myc-VCP. The control groups were transfected with the corresponding empty vectors. Post transfection (24 h), the cells were fixed and stained with anti-Myc (*green*) and anti-FLAG (*gray*) antibodies. The scale bar represents 20 μm. *E*, NS4B and VCP expression suppresses PKR-induced SG formation. HeLa cells were cotransfected with OSF-PKR (PKR-D328A), mCherry-G3BP, and a plasmid expressing either NS4B-Myc or Myc-VCP. Post transfection (24 h), the cells were fixed and stained with anti-FLAG and anti-Myc antibodies. The number of G3BP puncta and G3BP puncta–positive cells is indicated (*lower panel*). The protein expression levels were determined using anti-FLAG, anti-Myc tag, or anti-α-tubulin antibodies (*upper panels*). ∗∗*p* < 0.01 (Student’s *t* test). *F*, effect of DBeQ on NS4B-mediated suppression of PKR-induced SG formation. HeLa cells were cotransfected with OSF-PKR-D328A, mCherry-G3BP (*red*), and a plasmid expressing NS4B-Myc. Post transfection (24 h), the cells were treated with DBeQ for 4 h, then fixed and stained with anti-Myc (*green*) and anti-FLAG (*gray*) antibodies. The scale bar represents 20 μm. The numbers of cells containing G3BP dots among NS4B-positive cell population were plotted in the right graph. ∗∗*p* < 0.05 (Student’s *t* test). SG, stress granule; VCP, valosin-containing protein.

In flavivirus infection, the activated PKR-induced phosphorylation of eIF2α at S51 is reported to promote SG formation (18). Hence, the effect of PKR and eIF2α mutants on PKR-mediated SG induction, which is reported to be activated by dsRNAs, was examined (30). The cells were transfected with PKR-D328A (a constitutively active form capable of inducing eIF2α phosphorylation (31)), PKR-K296R (an unphosphorylated kinase-dead PKR), and eIF2α-S51D (a phosphomimetic mutant (32)). The overexpression of PKR-WT, PKR-D328A, and eIF2α-S51D promoted the formation of SGs (Fig. 8, *A* and *B*). In contrast, the overexpression of PKR-K296R, eIF2α-WT, or eIF2α-S51A did not promote SG formation (Fig. 8, *A* and *B*). These findings suggest that the formation of SGs in this study was mediated by PKR-dependent eIF2α phosphorylation. Next, the effect of NS4B or VCP expression on SG formation in PKR-D328A–expressing cells was examined. Although the expression of JEV–NS4B or VCP decreased the number of SGs, the expression level of PKR-D328A was similar to that in control cells (Fig. 8, *C*–*E*). These results suggest that NS4B and VCP impair the formation of SGs through PKR. Addition of the VCP inhibitor restored the formation of SGs in PKR-D328A–expressing cells even in the NS4B-expressing cells, suggesting that VCP mediated SG disassembly downstream of NS4B (Fig. 8*F*).

**Figure 9:**
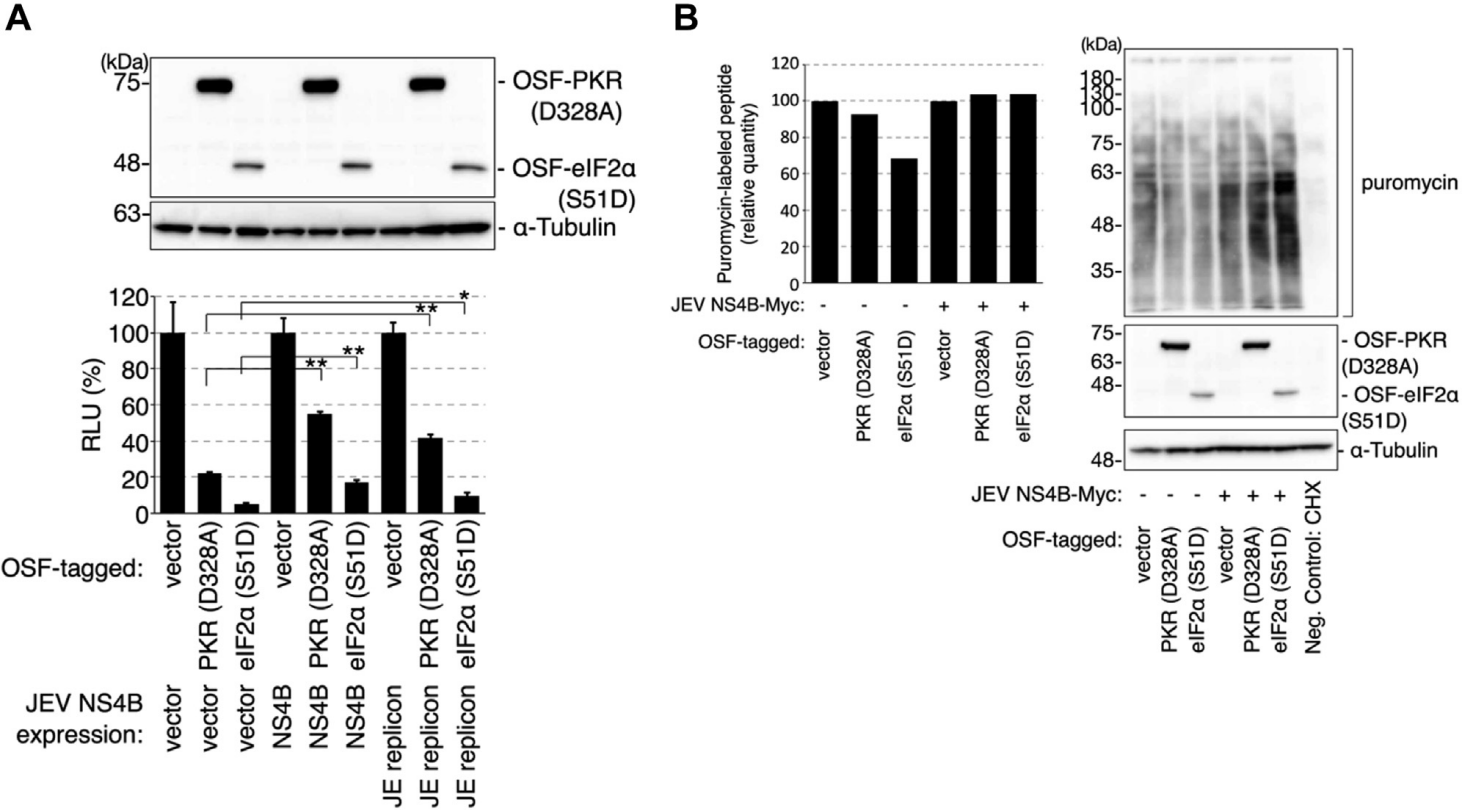
**Expression of NS4B impairs activated PKR–induced translational arrest.***A*, expression of NS4B impairs PKR-induced translational suppression. The 293T cells were cotransfected with cauliflower mosaic virus promoter-driven luciferase-expressing constructs, stress granule–inducing constructs (OSF-PKR-D328A or OSF-eIF2α-S51D), and JEV-NS4B-Myc or JEV subgenomic replicon constructs. Post transfection (24 h), the cells were lysed and the luciferase activities in the cells were measured (*lower graphs*). The OSF-PKR-D328A or OSF-eIF2α-S51D (*upper*) and α-tubulin (*middle*) protein levels were determined using the anti-FLAG and anti-α-tubulin antibodies, respectively. ∗∗*p* < 0.01 (Student’s *t* test). *B*, JEV-NS4B expression mitigated the PKR-D328A or eIF2α-S51D-induced translational arrest. Post transfection (24 h) with OSF-PKR-D328A or OSF-eIF2α-S51D vectors with or without JEV-NS4B-Myc vectors, the cells were treated with puromycin (5 μg/ml) for 25 min. The cells were harvested and lysed in a lysis buffer (1% Triton X-100, 150 mM NaCl, 50 mM Tris [pH 7.5], and proteinase inhibitors). Puromycin-bound proteins, OSF-PKR-D328A, OSF-eIF2α-S51D, α-tubulin were examined using Western blotting with the anti-puromycin, anti-FLAG, anti-α-tubulin antibodies. JEV, Japanese encephalitis virus; PKR, protein kinase R.

The inhibition of translation initiation is associated with SG formation. Moreover, SG formation was negatively correlated with viral replication and regulated by NS4B and VCP. Therefore, the effects of NS4B on PKR-mediated translational suppression were examined. The expression of PKR-D328A or eIF2α-S51D significantly suppressed the translation of luciferase reporter mRNA under the control of the cauliflower mosaic virus promoter, which is dependent on the 5′-cap structure (Fig. 9*A*, bottom panel, lanes 2 and 3). This translational inhibition was attenuated upon the expression of JEV-NS4B alone or transfection of JEV subgenomic replicon constructs (Fig. 9*A*). Furthermore, puromycin immunoblotting assay revealed that the global translational arrest was mitigated upon the expression of JEV-NS4B (Fig. 9*B*). Puromycin enters the A site of the ribosome. Therefore, the peptides were labeled with puromycin during synthesis. The translation activity was monitored by detecting the puromycin-bound elongating polypeptides. The puromycin signal intensity in the cells expressing PKR-D328A or eIF2α-S51D was lower than that in the cells transfected with a control vector (Fig. 9*B*, lanes 1–3). These results indicate that the expression of PKR-D328A or eIF2α-S51D inhibited the entire translation machinery. However, this inhibition was abolished upon the coexpression of NS4B (Fig. 9*B*, lanes 2–3 compared with lanes 5–6). Thus, NS4B has an inhibitory role in PKR-D328A or eIF2α-S51D–induced translational arrest. These results are consistent with our other findings on the importance of NS4B in mitigating eIF2α-mediated translational arrest and disassembling SGs. In summary, these results suggest that the NS4B–NPL4–VCP complex may function to resolve the SG to facilitate the translation of proteins required for viral replication under stress conditions.

## Discussion

**Figure 10:**
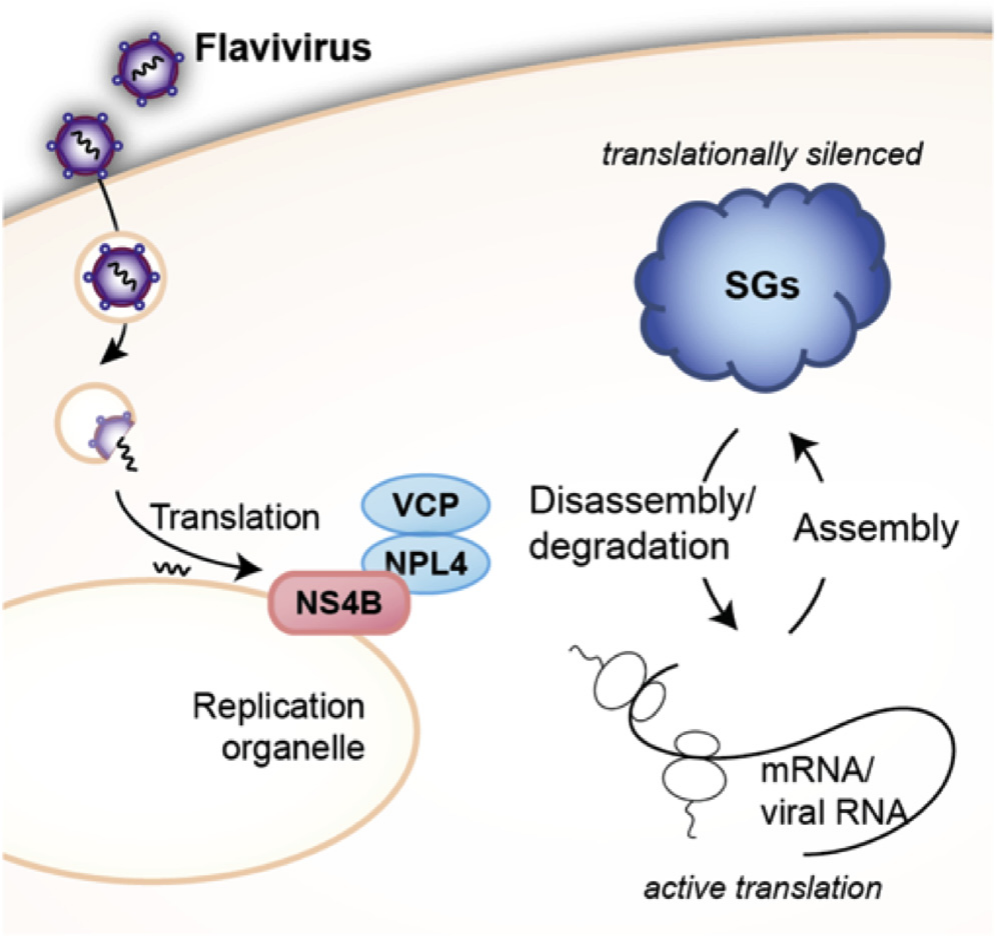
**Schematic presentation indicating the functions of NPL4–VCP complexes in flavivirus-infected cells.** The NPL4-VCP complexes are recruited to the viral replication organelle. This event may be important in the disassembly of SGs to facilitate efficient viral protein synthesis. SG, stress granule; VCP, valosin-containing protein.

This study demonstrated that the NPL4–VCP complex is an essential factor for JEV and DENV propagation by revealing a novel function of the NPL4–VCP complex, which involves the disassembly of SGs. Several viruses have evolved to evade various cellular antiviral responses and consequently promote self-replication. The findings of this study suggest that NS4B recruits VCP to evade the cellular stress responses by disassembling the SGs and facilitates the synthesis of proteins required for initiating viral genome replication (Fig. 10). These results are consistent with those of previous studies, which reported that ZIKV evades the host antiviral stress responses by modulating eIF2α dephosphorylation (23).

In the innate immune response, type I interferon induces the expression of PKR, which is activated by the dsRNA virus genome (18). Activated PKR inhibits cellular translational machinery by phosphorylating eIF2α (30) and consequently promotes the formation of cytoplasmic RNA granules (SGs). SGs are repeatedly assembled and disassembled during viral infection through PKR-mediated eIF2α phosphorylation and GADD34-mediated dephosphorylation, respectively (33). Flaviviruses do not markedly activate PKR (9, 34). However, the phosphorylation of eIF2α achieves a balance between acceleration and suppression of cellular protein synthesis. The findings of this study suggest that eIF2α-dependent translational activity can be regulated by SG assembly/disassembly, which is in turn regulated by the VCP complex and the phosphorylation status of eIF2α. However, SG formation is constitutively suppressed and PKR activity is upregulated in flavivirus-infected cells (9, 33). These previous findings suggest that the downstream PKR signaling is inhibited. This study demonstrated that the NPL4–VCP complex may play a role in the inhibition of PKR signaling. Viruses may activate PKR to inhibit cellular protein synthesis and recruit the VCP complex to promote viral protein synthesis. A similar scheme has been proposed in hepatitis C virus (HCV). HCV inhibits only eIF2α-mediated cellular protein synthesis by activating PKR and promotes eIF2α-independent IRES-mediated viral protein synthesis (35). A 3′ UTR-mediated 5′-cap–independent translation system has been proposed in flavivirus (36). However, VCP recruitment in flavivirus could have an additional or equivalent role in HCV-IRES–mediated stress-independent protein synthesis.

Several other mechanisms of inhibiting SG formation in flavivirus-infected cells have been reported. JEV or ZIKV capsid (a viral structural protein) directly interacts with caplin 1 (an SG component) and suppresses SG formation (20, 22). However, viral structural proteins are dispensable for flavivirus genome replication. In this study, VCP inhibition–mediated SG formation was observed in subgenomic replicon cells, which do not express capsid (Fig. 4*G*). This indicated that the VCP pathway is independent of capsid–caplin 1 interaction. The second mechanism involves the direct association of JEV-NS2A with PKR, which leads to the inhibition of PKR activation (37). However, this mechanism is not supported by the observations of our group and other groups, which reported that flavivirus suppresses oxidative stress–induced SG formation (9, 19, 29). The third mechanism involves the WNV-mediated upregulation and activation of transcription factors that modulate the antioxidant response, which leads to the impairment of arsenite-induced SG formation (21). Under oxidative stress conditions, this pathway may also have a role in anti-SG responses.

HRI is activated under oxidative stress conditions instead of PKR (38). NS4B expression impairs oxidative stress–induced SG formation (Fig. 7, *C* and *D*), which indicated that this inhibition machinery is not specific for the PKR pathway. Furthermore, a recent study demonstrated that DENV infection impairs not only eiF2α-dependent SG formation but also hippuristanol-induced eIF2α-independent SG formation. However, the authors suggest that the formation of SGs is not related to translational suppression (29). These results suggest that viruses regulate SG formation downstream of the pathway after SG formation. Flaviviruses may have multiple pathways to evade SG formation. The findings of this study are consistent with those of a previous study, which reported that WNV NS4B is involved in suppressing SG formation (9). This phenotype was confirmed in this study. However, NS4B expression alone was sufficient to suppress oxidative stress–induced SG formation (Fig. 7, *C* and *D*). The NPL4–VCP complex was associated with NS4B (Fig. 2) and SGs accumulated adjacent to the viral replication organelle in VCP-inhibited cells (Fig. 5). This suggests that the NS4B-mediated recruitment of the NPL4–VCP complex to the viral replication organelle facilitates viral protein synthesis by preventing SG formation.

The VCP complex is reported to be involved in other viral life cycles. In HCV and poliovirus infection, VCP has been identified as a cellular factor that facilitates viral genome replication (39, 40). The precise role of VCP in viral genome replication remains unclear. Yi *et al.* (40) reported that VCP is potentially involved in the assembly of the HCV replicase complex. VCP might also function as a counterpart for the stress response in these viral infections. Furthermore, we could not exclude the possibility that VCP is involved in the entry steps of viral infection. Phongphaew *et al.* (41) reported that WNV RNA level was downregulated even 2 h post infection in VCP-depleted cells, although the effect was minimal. Further experiments are required to clarify this question.

Previous studies have suggested that SG formation is limited in virus-infected cells as dsRNA (intermediate product of viral genome RNA processing) is masked in vesicles at the viral replication organelle (9, 19, 34). However, fluorescence analysis in this study demonstrated that SGs were formed adjacent to the viral replication organelle. A part of SG signals overlapped with the dsRNA signals located near CM (Fig. 5*A*). These results suggest that some parts of exposed dsRNA can be recognized by PKR and/or SG components. SGs comprise phase-separated RNA and RNA-binding proteins (42). Thus, SG signals are located at VP (an RNA replication site) but not at CM (a membrane protein accumulation site). The ubiquitin signals were also detected in the VP (Fig. 5*B*). Recently, G3BP1 ubiquitylation was reported to be a trigger for the disassembly of SGs (25). These observations suggest that NPL4-VCP–mediated SG disassembly processes are ongoing in this area. Of interest, the ubiquitin signals are also detected at the CM (Fig. 5*B*). These signals may indicate ubiquitylated NS proteins that can be degraded by ERAD at the CM (13). Furthermore, recent studies reported that the ULK1- and ULK2-mediated phosphorylation of VCP is required for the disassembly of SGs (43). ULK1/2 may regulate the NS4B-NPL4-VCP–mediated SG disassembly process.

Recently, various small compounds that specifically inhibit D1 and D2 ATPase activities have been developed (44, 45). These inhibitors are expected to be used as VCP-targeting therapeutics and can be potential candidates for developing antiflavivirus drugs.

## Experimental procedures

### Cells, viruses, and reagents

HeLa, 293A, 293T, Huh7, BHK-21, and Vero cells were cultured in Dulbecco’s modified Eagle’s medium supplemented with 10% fetal calf serum, 100 U/ml penicillin, and 100 μg/ml streptomycin at 5% CO_2_ and 37 °C. JEV AT31 and DENV 2 NGC strains (ATCC VR-1584) were cultured in 293A and BHK-21 cells, respectively. DBeQ (Sigma), Eer1 (Sigma), MDBN (StressMarq Biosciences), and rocaglamide A (Sigma) were dissolved in dimethyl sulfoxide. The culture medium of mock-treated cells contained the highest inhibitor concentration used in the respective assay. To induce the formation of SGs, the cells were cultured in a growth medium containing 100 μM sodium arsenite. The siRNAs, plasmids, and antibodies are listed in the Supplemental Information (Tables S1–S3). Virus titration was performed with a focus forming assay as described (46). For the infection study, viruses were inoculated at a multiplicity of infection of 0.3 for propagation and 1.0 for imaging experiments, including the quantification of SGs.

### siRNA-mediated knockdown and rescue experiments

siRNA-mediated gene silencing and gene rescue experiments were performed as described (46). Briefly, HEK293A cells transfected twice with 10 nM siRNA using Lipofectamine RNAiMAX transfection reagent (Thermo Fisher Scientific) at 24 h intervals were infected with viruses 24 h post transfection. The culture supernatant was collected 48 h post infection. The viral titer in the culture supernatant was measured using a focus forming assay. For rescue experiments, siRNAs were mixed with plasmids encoding VCP with a silence mutation at the siRNA target sequence (see Table S1). The cells were transfected with this mixture using Lipofectamine 3000 (Thermo Fisher Scientific).

### Immunofluorescence

Cells cultured on glass coverslips were fixed with 4% paraformaldehyde in PBS (Nacalai Tesque) for 10 min, permeabilized with PBS containing 0.1% Triton X-100, blocked with 10% fetal bovine serum, and incubated with the diluted primary antibodies (see Table S2) for 60 min at 25 °C. Next, the cells were incubated with various Alexa Fluor–conjugated secondary antibodies in PBS for 60 min. The cells were then mounted on coverslips with Fluoromount-G (SouthernBiotech). The images were captured using an Olympus FV3000 laser scanning confocal microscope.

### Y2H analysis

Directed two-hybrid assays were performed using the Matchmaker GAL4 Yeast Two-Hybrid 3 system (Clontech). The pGADT7-based and pGBKT7-based plasmids (see Table S3) were cotransformed into the competent yeast strain AH109, following the manufacturer’s instructions. The transformation efficiencies were tested using control plates (Leu/Trp deficient). The interactions were examined using selective plates (Leu/Trp/Ade/His deficient).

### Measurement of luciferase activity

NanoLuc luciferase and firefly luciferase activities were measured using the Nano-Glo Luciferase Assay System (Promega) and Bright-Glo Luciferase Assay System (Promega), respectively. The cells were washed with PBS and lysed in a lysis buffer (1% Triton-X100, 50 mM Tris-HCl pH 7.4, 150 mM NaCl, and protease inhibitors [Roche]). The cleared lysate was mixed with luciferase assay buffer (Promega). The luminesce intensity of the mixture was measured using a luminometer (Fluoroskan Ascent FL, Labsystems) for 10 s. Values were represented as relative light units.

### Strep-Tactin pull-down

Post transfection (48 h), HEK293T cells were lysed in lysis buffer (150 mM NaCl, 20 mM Tris-HCl [pH 7.5], and 1% Triton X-100) supplemented with complete protease inhibitor cocktail (Roche). The cell lysates were centrifuged at 20,000*g* and 4 °C for 10 min. The supernatant was incubated with Strep-Tactin Sepharose beads (IBA GmbH) for 1 h at 4 °C. The beads were washed four times with wash buffer (150 mM NaCl, 20 mM Tris-HCl [pH 7.5], and 0.1% Triton X-100) and incubated with Laemmli sample buffer (125 mM Tris-HCl [pH 6.7], 20% glycerol, 0.01% bromophenol blue, 10% 2-mercaptoethanol, and 4% SDS) for 5 min at 95 °C. The input lysate and eluate were then subjected to Western blotting.

### Immunoprecipitation for endogenous proteins

Protein A beads were washed thrice with wash buffer and incubated with the anti-JEV-NS4B antibody at 4 °C for 1 h. After antibody conjugation, the beads were rewashed thrice with the wash buffer. HEK293T cells were collected after infection with JEV at MOI = 1.0 for 48 h. The cells were washed twice with ice-cold PBS and lysed in lysis buffer supplemented with a complete protease inhibitor cocktail (Roche) on ice for 5 min. The cell lysates were cleared *via* centrifuging at 20,000*g* and 4 °C for 10 min. The lysates were incubated with the anti-JEV-NS4B antibody–conjugated protein A beads at 4 °C for 1 h. The beads were then washed thrice with wash buffer and incubated with Laemmli sample buffer for 5 min at 95 °C. The input lysate and eluate were then subjected to Western blotting.

### Pull-down HiBiT assay

The cells were washed twice with ice-cold PBS and lysed in lysis buffer supplemented with a complete protease inhibitor cocktail (Roche), on ice for 5 min. The cell lysates were centrifuged at 20,000*g* and 4 °C for 10 min, and HiBiT-dependent luciferase activity (HiBiT activity) was measured in the input fraction using the Nano Glo HiBiT Lytic Detection System (Promega). The Strep-Tactin pull-down protocol was followed; briefly, the beads were washed four times with the wash buffer, suspended in destiobiotin-containing 1 × elution buffer (IBA), and incubated at 4 °C for 1 h. The eluted fractions were collected *via* centrifugation at 20,000*g* and 4 °C for 1 min, and HiBiT activity was measured. Subsequently, the collected Strep-Tactin elution fraction was incubated with the anti-Myc antibody–conjugated protein A beads at 4 °C for 1 h. The beads were washed thrice with wash buffer and resuspended in the wash buffer. Then, HiBiT activity in the bead-bound fraction was measured.

### Puromycin labeling assay

HEK293T cells were transfected with plasmid using polyethylenimine (PEI). PEI (Polysciences) was mixed with plasmids at a ratio of 3:1 (w/w) and incubated at room temperature for 15 min. The DNA/PEI mixture was added to the cell culture medium. Post transfection (24 h), the cells were treated with 5 μg/ml puromycin for 25 min and lysed in lysis buffer. The cell lysate was centrifuged at 20,000*g* and 4 °C for 10 min. The puromycin-labeled peptides in the clarified lysate were detected using Western blotting with anti-puromycin antibodies.

### Western blotting and HiBiT blotting

The cells were washed with ice-cold PBS, scraped, collected by centrifugation at 500*g* and 4 °C for 10 min, and lysed in lysis buffer. The lysates were mixed with 2 × Laemmli sample buffer and subjected to SDS-PAGE. The resolved proteins were transferred to an Immobilon-P polyvinylidene difluoride membrane (Millipore). The membrane was blocked with blocking buffer (3% or 0.3% skim milk in TBS-T [25 mM Tris (pH 7.5), 137 mM NaCl, 0.27 mM KCl, and 0.05% Tween 20]) for 30 min and incubated with the primary antibodies (see Table S2) at 4 °C overnight. Next, the membrane was incubated with secondary antibodies at room temperature for 50 min. Immunoreactive signals were developed using EzWestLumi plus (ATTO) and observed using the iBright Imaging System (Thermo Fisher Scientific). For HiBiT blotting, the protein-bound Immobilon-P polyvinylidene difluoride membrane was treated with the bacterially expressed recombinant LgBiT and NanoLuc substrate of the Nano-Glo Luciferase Assay System (Promega). The luminescence signals were observed using the Molecular Imager iBright Imaging System.

### DNA-based subgenomic replicon analysis

The type 1 dengue (DENV1) subgenomic reporter replicon and RdRp-inactivated subgenomic reporter replicon-expressing plasmid DNA, designated pCMV (Δ4.5p)-D1-nluc-rep and pCMV (Δ4.5p)-D1-nluc-rep-fs, respectively, were used in this study. These plasmids were constructed from previously described plasmids, pCMV-D1-nluc-rep and pCMV-D1-nluc-rep-fs, respectively (47), by deleting the extensive region of the CMV promoter. Briefly, after a 24-h transfection of the 293T cells described above with the indicated siRNAs, the cells were transfected with each replicon plasmid DNA using Lipofectamine 3000 (Thermo fisher). After 72 h of post-plasmid DNA transfection, NanoLuc luciferase activity was determined in cells using the Nano-Glo luciferase assay system and the Varioskan LUX Multimode Microplate Reader (Thermo fisher Scientific) according to the manufacturer's instructions. For Western blot sample preparation, the cells were directly lysed in Laemmli’s sample buffer.

### Statistical analysis

The means between two groups were compared using the Student’s *t* test. Differences were considered significant at ∗*p* < 0.05 and ∗∗*p* < 0.01. Additional methods, extended data display, and discussion are available in Supplemental Information.

## Data availability

The data that support the findings of this study are available from the corresponding author, Eiji Morita, upon reasonable request.

## Supporting information

This article contains supporting information.

## Conflict of interest

The authors declare that they have no conflicts of interest with the contents of this article.

## References

[1] Gould E., Solomon T. (2008). Pathogenic flaviviruses. Lancet.

[2] Chambers T.J., Hahn C.S., Galler R., Rice C.M. (1990). Flavivirus genome organization, expression, and replication. Annu. Rev. Microbiol..

[3] Kümmerer B.M., Rice C.M. (2002). Mutations in the yellow fever virus nonstructural protein NS2A selectively block production of infectious particles. J. Virol..

[4] Leung J.Y., Pijlman G.P., Kondratieva N., Hyde J., Mackenzie J.M., Khromykh A.A. (2008). Role of nonstructural protein NS2A in flavivirus assembly. J. Virol..

[5] Miller S., Kastner S., Krijnse-Locker J., Bühler S., Bartenschlager R. (2007). The non-structural protein 4A of dengue virus is an integral membrane protein inducing membrane alterations in a 2K-regulated manner. J. Biol. Chem..

[6] Kaufusi P.H., Kelley J.F., Yanagihara R., Nerurkar V.R. (2014). Induction of endoplasmic reticulum-derived replication-competent membrane structures by West Nile virus non-structural protein 4B. PLoS One.

[7] Roosendaal J., Westaway E.G., Khromykh A., Mackenzie J.M. (2006). Regulated cleavages at the West Nile virus NS4A-2K-NS4B junctions play a major role in rearranging cytoplasmic membranes and Golgi trafficking of the NS4A protein. J. Virol..

[8] Chatel-Chaix L., Fischl W., Scaturro P., Cortese M., Kallis S., Bartenschlager M., Fischer B., Bartenschlager R. (2015). A combined genetic-proteomic approach identifies residues within dengue virus NS4B critical for interaction with NS3 and viral replication. J. Virol..

[9] Courtney S.C., Scherbik S.V., Stockman B.M., Brinton M.A. (2012). West Nile virus infections suppress early viral RNA synthesis and avoid inducing the cell stress granule response. J. Virol..

[10] Zmurko J., Neyts J., Dallmeier K. (2015). Flaviviral NS4b, chameleon and jack-in-the-box roles in viral replication and pathogenesis, and a molecular target for antiviral intervention. Rev. Med. Virol..

[11] Meyer H., Bug M., Bremer S. (2012). Emerging functions of the VCP/p97 AAA-ATPase in the ubiquitin system. Nat. Cell Biol..

[12] Meyer H., Weihl C.C. (2014). The VCP/p97 system at a glance: Connecting cellular function to disease pathogenesis. J. Cell Sci..

[13] Tabata K., Arakawa M., Ishida K., Kobayashi M., Nara A., Sugimoto T., Okada T., Mori K., Morita E. (2021). Endoplasmic reticulum-associated degradation controls virus protein homeostasis, which is required for flavivirus propagation. J. Virol..

[14] Sehrawat S., Khasa R., Deb A., Prajapat S.K., Mallick S., Basu A., Surjit M., Kalia M., Vrati S. (2021). Valosin-containing protein/p97 plays critical roles in the Japanese encephalitis virus life cycle. J. Virol..

[15] Ramanathan H.N., Zhang S., Douam F., Mar K.B., Chang J., Yang P.L., Schoggins J.W., Ploss A., Lindenbach B.D. (2020). A sensitive yellow fever virus entry reporter identifies valosin-containing protein (VCP/p97) as an essential host factor for flavivirus uncoating. mBio.

[16] Chan Y.K., Gack M.U. (2016). Viral evasion of intracellular DNA and RNA sensing. Nat. Rev. Microbiol..

[17] Panas M.D., Ivanov P., Anderson P. (2016). Mechanistic insights into mammalian stress granule dynamics. J. Cell Biol..

[18] Miller C.L. (2011). Stress granules and virus replication. Future Virol..

[19] Emara M.M., Brinton M.A. (2007). Interaction of TIA-1/TIAR with West Nile and dengue virus products in infected cells interferes with stress granule formation and processing body assembly. Proc. Natl. Acad. Sci. U. S. A..

[20] Katoh H., Okamoto T., Fukuhara T., Kambara H., Morita E., Mori Y., Kamitani W., Matsuura Y. (2013). Japanese encephalitis virus core protein inhibits stress granule formation through an interaction with caprin-1 and facilitates viral propagation. J. Virol..

[21] Basu M., Courtney S.C., Brinton M.A. (2017). Arsenite-induced stress granule formation is inhibited by elevated levels of reduced glutathione in West Nile virus-infected cells. PLoS Pathog..

[22] Hou S., Kumar A., Xu Z., Airo A.M., Stryapunina I., Wong C.P., Branton W., Tchesnokov E., Götte M., Power C., Hobman T.C. (2017). Zika virus hijacks stress granule proteins and modulates the host stress response. J. Virol..

[23] Amorim R., Temzi A., Griffin B.D., Mouland A.J. (2017). Zika virus inhibits eIF2α-dependent stress granule assembly. PLoS Negl. Trop. Dis..

[24] Buchan J.R., Kolaitis R.-M., Taylor J.P., Parker R. (2013). Eukaryotic stress granules are cleared by autophagy and Cdc48/VCP function. Cell.

[25] Gwon Y., Maxwell B.A., Kolaitis R.-M., Zhang P., Kim H.J., Taylor J.P. (2021). Ubiquitination of G3BP1 mediates stress granule disassembly in a context-specific manner. Science.

[26] Chou T.-F., Brown S.J., Minond D., Nordin B.E., Li K., Jones A.C., Chase P., Porubsky P.R., Stoltz B.M., Schoenen F.J., Patricelli M.P., Hodder P., Rosen H., Deshaies R.J. (2011). Reversible inhibitor of p97, DBeQ, impairs both ubiquitin-dependent and autophagic protein clearance pathways. Proc. Natl. Acad. Sci. U. S. A..

[27] Wang Q., Shinkre B.A., Lee J., Weniger M.A., Liu Y., Chen W., Wiestner A., Trenkle W.C., Ye Y. (2010). The ERAD inhibitor Eeyarestatin I is a bifunctional compound with a membrane-binding domain and a p97/VCP inhibitory group. PLoS One.

[28] Seguin S.J., Morelli F.F., Vinet J., Amore D., De Biasi S., Poletti A., Rubinsztein D.C., Carra S. (2014). Inhibition of autophagy, lysosome and VCP function impairs stress granule assembly. Cell Death Differ..

[29] Roth H., Magg V., Uch F., Mutz P., Klein P., Haneke K., Lohmann V., Bartenschlager R., Fackler O.T., Locker N., Stoecklin G., Ruggieri A. (2017). Flavivirus infection uncouples translation suppression from cellular stress responses. mBio.

[30] Taylor S.S., Haste N.M., Ghosh G. (2005). PKR and eIF2α: Integration of kinase dimerization, activation, and substrate docking. Cell.

[31] Li S., Peters G.A., Ding K., Zhang X., Qin J., Sen G.C. (2006). Molecular basis for PKR activation by PACT or dsRNA. Proc. Natl. Acad. Sci. U. S. A..

[32] Perkins D.J., Barber G.N. (2004). Defects in translational regulation mediated by the α subunit of eukaryotic initiation factor 2 inhibit antiviral activity and facilitate the malignant transformation of human fibroblasts. Mol. Cell. Biol..

[33] Ruggieri A., Dazert E., Metz P., Hofmann S., Bergeest J.-P., Mazur J., Bankhead P., Hiet M.-S., Kallis S., Alvisi G., Samuel C.E., Lohmann V., Kaderali L., Rohr K., Frese M. (2012). Dynamic oscillation of translation and stress granule formation mark the cellular response to virus infection. Cell Host Microbe.

[34] Elbahesh H., Scherbik S.V., Brinton M.A. (2011). West Nile virus infection does not induce PKR activation in rodent cells. Virology.

[35] Garaigorta U., Chisari F.V. (2009). Hepatitis C virus blocks interferon effector function by inducing protein kinase R phosphorylation. Cell Host Microbe.

[36] Edgil D., Polacek C., Harris E. (2006). Dengue virus utilizes a novel strategy for translation initiation when cap-dependent translation is inhibited. J. Virol..

[37] Tu Y.-C., Yu C.-Y., Liang J.-J., Lin E., Liao C.-L., Lin Y.-L. (2012). Blocking double-stranded RNA-activated protein kinase PKR by Japanese encephalitis virus nonstructural protein 2A. J. Virol..

[38] Xu Z., Pal J.K., Thulasiraman V., Hahn H.P., Chen J.-J., Matts R.L. (1997). The role of the 90-kDa heat-shock protein and its associated cohorts in stabilizing the heme-regulated eIF-2alpha kinase in reticulocyte lysates during heat stress. Eur. J. Biochem..

[39] Arita M., Wakita T., Shimizu H. (2012). Valosin-containing protein (VCP/p97) is required for poliovirus replication and is involved in cellular protein secretion pathway in poliovirus infection. J. Virol..

[40] Yi Z., Fang C., Zou J., Xu J., Song W., Du X., Pan T., Lu H., Yuan Z. (2016). Affinity purification of the hepatitis C virus replicase identifies valosin-containing protein, a member of the ATPases associated with diverse cellular activities family, as an active virus replication modulator. J. Virol..

[41] Phongphaew W., Kobayashi S., Sasaki M., Carr M., Hall W.W., Orba Y., Sawa H. (2017). Valosin-containing protein (VCP/p97) plays a role in the replication of West Nile virus. Virus Res..

[42] Wheeler J.R., Matheny T., Jain S., Abrisch R., Parker R. (2016). Distinct stages in stress granule assembly and disassembly. Elife.

[43] Wang B., Maxwell B.A., Joo J.H., Gwon Y., Messing J., Mishra A., Shaw T.I., Ward A.L., Quan H., Sakurada S.M., Pruett-Miller S.M., Bertorini T., Vogel P., Kim H.J., Peng J. (2019). ULK1 and ULK2 regulate stress granule disassembly through phosphorylation and activation of VCP/p97. Mol. Cell.

[44] Ikeda H.O., Sasaoka N., Koike M., Nakano N., Muraoka Y., Toda Y., Fuchigami T., Shudo T., Iwata A., Hori S., Yoshimura N., Kakizuka A. (2015). Novel VCP modulators mitigate major pathologies of rd10, a mouse model of retinitis pigmentosa. Sci. Rep..

[45] Chou T.-F., Deshaies R.J. (2011). Development of p97 AAA ATPase inhibitors. Autophagy.

[46] Tabata K., Arimoto M., Arakawa M., Nara A., Saito K., Omori H., Arai A., Ishikawa T., Konishi E., Suzuki R., Matsuura Y., Morita E. (2016). Unique requirement for ESCRT factors in flavivirus particle formation on the endoplasmic reticulum. Cell Rep..

[47] Matsuda M., Yamanaka A., Yato K., Yoshii K., Watashi K., Aizaki H., Konishi E., Takasaki T., Kato T., Muramatsu M., Wakita T., Suzuki R. (2018). High-throughput neutralization assay for multiple flaviviruses based on single-round infectious particles using dengue virus type 1 reporter replicon. Sci. Rep..

